# Association between Socioecological Status, Nutrient Intake, and Cancer Screening Behaviors in Adults Aged 40 and Over: Insights from the Eighth Korea National Health and Nutrition Examination Survey (KNHANES, 2019)

**DOI:** 10.3390/nu16071048

**Published:** 2024-04-03

**Authors:** Seungpil Jeong, Yean-Jung Choi

**Affiliations:** 1 Department of Medical Informatics, College of Medicine, Catholic University of Korea, Seoul 06591, Republic of Korea; seungpil720@catholic.ac.kr; 2 Department of Food and Nutrition, Sahmyook University, Seoul 01795, Republic of Korea

**Keywords:** cancer screening, socioecology, nutritional status, KNHANES, lifestyle

## Abstract

Cancer screening is pivotal for early detection and improved survival rates. While socio-ecological factors are known to influence screening uptake, the role of lifestyle, dietary habits, and general health in shaping these decisions remains underexplored. Utilizing the 2019 Korea National Health and Nutrition Examination Survey (KNHANES), this study examined the myriad of factors impacting cancer screening utilization. Data from 274,872 adults aged 40 years or older were scrutinized, highlighting demographics, income, lifestyle behaviors, health-related variables, nutrient intake, and dietary quality. A combination of descriptive statistics and logistic regression helped us ascertain influential determinants. Higher educational attainment and income quartiles were positively correlated with cancer screening rates. Regular walkers, those engaged in moderate physical activity, and individuals with a previous cancer diagnosis were more likely to get screened. High-risk drinkers and smokers were less inclined towards screening. Dietary habits also influenced screening decisions. Notably, participants with healthier eating behaviors, indicated by factors such as regular breakfasts and fewer meals out, were more likely to undergo screening. Additionally, nutrient intake analysis revealed that those who had undergone screening consumed greater quantities of most nutrients, bar a few exceptions. For individuals aged 50–64, nutritional assessment indicators highlighted a higher mean adequacy ratio (MAR) and index of nutritional quality (INQ) value among those who participated in screening, suggesting better nutritional quality. This study elucidates the complex socio-ecological and nutritional landscape influencing cancer screening decisions. The results underscore the importance of a holistic approach, emphasizing lifestyle, dietary habits, and socio-economic considerations. It provides a roadmap for policymakers to craft more inclusive screening programs, ensuring equal access and promoting early detection.

## 1. Introduction

Cancer continues to be a predominant health concern worldwide [1,2]. Despite 25 years of concerted efforts under the National Cancer Control Plan (NCCP), cancer’s menace remains undiminished as a leading cause of mortality [3,4,5]. National statistics on cancer incidence from 1999 reveal an annual average increase of 3.5% in the age-standardized incidence rate for all cancers from 1999 to 2012. This trajectory changed from 2012 to 2015, recording an annual average decline of −5.4%. However, post 2015, the trend remained relatively static [6]. As of 2020, the crude incidence rate of cancer is 496.2 per 100,000 population [7]. An age-wise analysis shows that those aged 65 and older have an alarming rate of 1483.6 per 100,000, underscoring the pronounced vulnerability of the elderly demographic [6].

This alarming epidemiological landscape underscores the crucial role of early diagnosis in cancer control, as emphasized by the World Health Organization (WHO). The success of early detection strategies in East Asian countries, especially with high survival rates in early-stage gastric cancer, exemplifies the life-saving potential of early diagnosis and the urgent need to enhance these detection methods globally; in fact, over half of gastric cancer cases are identified at the early tumor stage, leading to a 5-year survival rate of over 90% [8,9,10,11].

Understanding the context of healthcare systems is crucial in discussing cancer control strategies. South Korea, for example, has a universal healthcare system, known as the National Health Insurance Service (NHIS). This system provides comprehensive medical coverage to almost all residents in the country, funded through a combination of citizen contributions, government subsidies, and tobacco surcharges. While the public healthcare system is not free, it offers affordable coverage of 50–80% of medical costs, depending on the treatment. Despite this universal coverage, a significant proportion of South Koreans also opt for private health insurance to cover additional costs, such as copays or service fees [12].

Building on this, many studies have shown that persistent inequalities in screening rates across different economic groups and the significant impact of screening costs highlight the complex interplay of socioeconomic status and health behaviors in cancer prevention. While socioeconomic status and positive health behaviors have been identified as major predictors of cancer screening participation [13,14,15], there exist disparities based on economic capacity [16]. The Republic of Korea, in its bid to enhance screening access, introduced the National Early Cancer Screening Project for economically disadvantaged families in 1999 [17]. Nevertheless, disparities persist [18,19]. Previous findings indicate differences in screening rates between self-pay screenings and those facilitated by the National Health Insurance Service [20,21]. The cost implication has, unsurprisingly, been identified as a barrier, with higher costs correlating to lower screening rates [22,23,24,25,26,27,28].

In light of these considerations, our study aims to explore the intricate relationship between socioecological status, nutrient intake, and cancer screening behaviors among adults aged 40 and over in Korea. We hypothesize that socioecological status and nutrient intake significantly influence the likelihood of participating in cancer screening programs. Specifically, our research anticipates uncovering a correlation where individuals with lower socioecological status and inadequate nutrient intake demonstrate reduced engagement in cancer screening activities. This hypothesis is grounded in the understanding that socioecological factors, including economic and educational backgrounds, as well as dietary habits, play a crucial role in shaping health behavior and access to preventive healthcare services. By examining these associations, our study seeks to provide insights into how disparities in social and nutritional aspects can impact the preventive health practices, particularly in the context of cancer screening, among the middle-aged and older population in Korea.

An intriguing observation from past studies has been the bias in the adoption of self-pay cancer screenings by high-income groups, while the free screenings without co-payment have been predominantly accessed by those in lower income brackets [14,29,30]. However, relying solely on free screenings might prove inadequate for comprehensive early cancer detection. We delve into a critical yet underexplored aspect: the socioeconomic biases in the adoption of self-pay cancer screenings and the reliance on free screenings by lower income groups. This observation raises concerns about the adequacy of current screening strategies and highlights a significant gap in research, particularly the lack of studies exploring the potential correlation between dietary habits and cancer screening behaviors. This gap serves as the foundation for our study. Research focusing on disparities in the use of preventive medical services, like cancer screenings, based on income levels is scarce [19,24,25,26,27]. Moreover, no study to date has probed the potential correlation between dietary habits, a significant social determinant, and cancer screening behaviors [31,32,33].

In this backdrop, our study sought to bridge this knowledge gap by analyzing national-level data from the 2019 Korea National Health and Nutrition Examination Survey (KNHANES). We aimed to discern the socio-ecological and nutritional status influencing cancer screening behaviors. Additionally, this study endeavored to illuminate the relationship between the quality of meals and cancer screening uptake.

## 2. Methods

### 2.1. Data Source and Research Objectives

In this study, our primary objectives were to explore the relationship between socio-ecological status and cancer screening behaviors, analyze the impact of nutritional status on cancer screening participation, and examine the interplay between socio-demographic factors, nutrient intakes, and the different types of cancer screenings, which include self-pay, partial-pay, and free screenings. For this purpose, we utilized the data from the KNHANES as provided by the Korea Centers for Disease Control and Prevention.

The KNHANES conducts an annual survey by selecting 25 households from each of 192 regions as a representative probability sample, surveying approximately 10,000 household members aged one and above. Participants are divided into three age groups-children (1–11 years old), adolescents (12–18 years old), and adults (19 years and older), with specific survey items tailored to each group’s lifecycle characteristics. In each survey district, a professional survey team conducts assessments over three days, with a mobile examination vehicle facilitating checkups and health surveys, usually operating from 6 a.m. to 1 p.m. The process for each adult takes about 1 h 30 min to 2 h. The examination includes checking for chronic diseases such as obesity, high blood pressure, diabetes, and dyslipidemia through height, weight, blood pressure measurements, blood and urine tests, and oral examinations. The health survey investigates behaviors such as smoking, drinking, physical activity, and mental health through questionnaires using computer-assisted personal interviewing (CAPI) technology. The questionnaire used has been validated in previous KNHANES cycles. The nutritional survey is comprehensive, involving the assessment of dietary habits and types and amounts of food consumed. This systematic approach allows KNHANES to provide a rich dataset for understanding the health and nutritional status across various demographics in Korea.

The management and storage of the KNHANES data were conducted in strict compliance with ethical guidelines and with the approval of our institution’s review board. The data were used exclusively for the purposes of this study and were stored securely, adhering to the relevant data protection regulations. Any requests for data sharing or secondary analysis of the KNHANES data will be subject to review and approval by our institution’s ethical review board. The KNHANES data, being publicly available for research purposes, is anonymized to protect the privacy of the participants. Details regarding the duration of data storage and the specific data management protocols are in accordance with the guidelines provided by the Korea Centers for Disease Control and Prevention.

**Figure 1:**
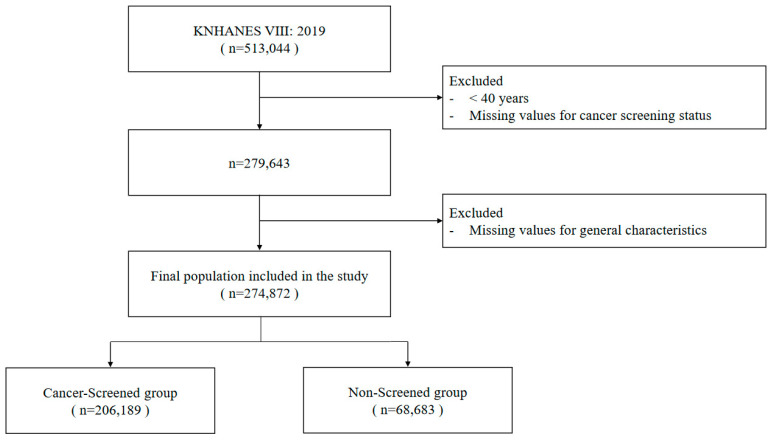
Flowchart of the participant selection process for the study.

Cancer screenings were categorized into three main types: self-pay, partial-pay, and free screening. This categorization helped elucidate the prevailing trends of cancer screening by payment type. Of the 513,044 participants in the 2019 KNHANES, 279,643 were aged 40 or above, the age recommended for cancer screening. After accounting for non-responses to key items and missing values, the final sample comprised 274,872 individuals, yielding a response rate of 98.29% (Figure 1).

### 2.2. Variable Descriptions

#### 2.2.1. Dependent Variable

The study’s primary outcome, or the dependent variable, was the cancer screening status of adults aged 40 and above. Responses to the question, “Have you undergone cancer screening in the past 2 years?” from the survey determined this variable. Screenings were further characterized by self-pay cancer screening (comprehensive health assessments and cancer screenings undertaken at hospitals, clinics, or comprehensive screening facilities), partial self-pay cancer screening (specific screenings, where costs are partially covered by the patient), and national health insurance cancer screening (screenings fully subsidized by the National Health Insurance Corporation or those available for free at public health centers).

#### 2.2.2. Socio-Demographic Variables

This study considered various socio-demographic factors, such as gender, age, marital status, educational background, residential location, economic status, household income, insurance category, and the possession of private insurance. Following the National Cancer Screening Guidelines, age brackets were set as 40–49, 50–64, and 65 and above, in alignment with the Dietary Reference Intakes for Koreans 2020 used for assessing dietary behavior. Marital status categories were ‘with spouse’ and ‘without spouse’, the latter combining individuals who were divorced, widowed, separated, or never married. Educational attainment was divided into elementary or below, middle school, high school, and junior college or higher. Places of residence were classified as Seoul, Gyeonggi, one of the six metropolitan cities, or other regions. Household income was segmented into quartiles, termed as low, lower middle, upper middle, and high. For insurance, individuals under medical benefit types 1 and 2 were grouped as medical benefit recipients, while others with local or employment-based insurance were designated as health insurance subscribers.

#### 2.2.3. Lifestyle and Health-Related Variables

Lifestyle and health variables encompassed subjective health perception, stress levels, smoking habits, alcohol consumption, moderate physical activity, cancer history, and chronic disease count. Subjective health was categorized as good (either very good or good), average, or poor (either bad or very bad). Stress perception was divided into ‘low’ or ‘high’. Drinking was categorized as ‘non-drinker’ for those who never consumed alcohol or consumed less than one drink per month in the past year and ‘drinker’ for those consuming more than one drink per month in the previous year. Smoking was categorized into ‘current smoker’ (regular or occasional) and ‘non-smoker’ (former or never smoked). Responses to engagement in moderate-intensity physical activities that cause mild breathlessness or a slightly elevated heartbeat for a minimum of 10 min were categorized as ‘yes’ or ‘no’. For this study’s purposes, this was referred to as physical activity.

The presence or absence of specific cancers was determined by physician diagnoses, including, but not limited to, gastric, liver, and colon cancers. Chronic disease count covered a list of 18 diseases, such as hypertension, dyslipidemia, and diabetes. Participants’ affirmative responses determined the count, which was then grouped into 0, 1, 2, or 3 and above. The Charlson Comorbidity Index (CCI) scores were computed as described by Gang and colleagues [34] and were divided into ‘0’, ‘1’, and ‘≥2’.

### 2.3. Dietary Intake Assessment

The KNHANES nutritional survey encompasses dietary habits, food frequency, and actual consumption, gathered via direct interviews [35]. This is achieved through a detailed process, which includes gathering information on meals and specific food items, estimating the weight or volume of each food item using tools like measuring cups and spoons, classifying items for further investigation (including types and brands of seasonings used), confirming the accuracy of collected food information, and conducting supplementary surveys for comprehensive dietary data. In our study, nutrient intake was derived from this dataset, covering 18 nutrients, inclusive of energy, protein, fat, and carbohydrates. The food intake survey employed a 24 h recall method, facilitating open-ended responses for diverse meals and foods consumed. To assess nutrient adequacy or excess based on age and gender, we referred to the 2020 Dietary Reference Intakes for Koreans [36].

The nutrient adequacy ratio (NAR) was determined for each nutrient, capped at 1 if the ratio exceeded this value. The mean adequacy ratio (MAR) represents the NAR average, reflecting the holistic quality of nutrient consumption. The index of nutritional quality (INQ) gauges nutrient balance in a meal, calculated as the nutrient intake per 1000 kcal divided by the recommended intake for equivalent energy expenditure. An INQ of 1 or above signifies nutrient adequacy concerning energy intake guidelines [37].

### 2.4. Statistical Analysis

All data underwent statistical analysis in the SAS (version 9.4; SAS Institute, Cary, NC, USA) software. Variables were analyzed considering the composite sample design data method, accounting for clustering, stratification, and weights from the 8th KNHANES. Descriptive statistics, including height, weight, waist circumference, and BMI, were articulated as mean ± standard deviation. To assess the normality of our data, we employed the Kolmogorov–Smirnov test, a well-established method particularly effective for large sample sizes. Additionally, we supplemented this analysis by inspecting Q-Q plots and histograms, which provided visual confirmation of the distribution characteristics of our dataset. Participants were grouped based on their cancer screening status and household income, with the chi-square test applied to categorical variables. The ANCOVA and Scheffe post hoc tests were employed for continuous variables. Multiple logistic regression, adjusted for age and gender, assessed relationships between independent variables and cancer screening status. Variances in daily nutrient intake based on screening status were analyzed using generalized linear models (GLMs). GLMs are a flexible extension of ordinary linear regression that allows for response variables to have error distribution models other than a normal distribution. GLMs are particularly useful in dealing with categorical outcomes like screening status as they can model binary or multinomial outcomes using logistic or multinomial regression [38,39,40,41]. We chose to use GLMs due to their ability to handle the binary nature of our dependent variable (cancer screening status: screened or not screened) and to accommodate the distribution of our independent variables. This approach allowed us to robustly model the relationship between socio-demographic, lifestyle, and nutritional factors and the likelihood of undergoing cancer screening. Lastly, the relationship between cancer screening status and daily nutrient intake was evaluated using multiple logistic regression, adjusted for age and gender. A *p*-value of <0.05 was considered statistically significant.

## 3. Results

### 3.1. Characteristics of the Study Participants

Table 1 details the distribution of participants based on age, sex, marital status, economic activity, regional residence, education level, household income, health insurance type, and private insurance subscription. Notably, each of these variables demonstrated statistically significant differences in cancer screening rates among the subjects.

When analyzing cancer screening rates by age, the 40–49 and 50–64 age groups exhibited similar rates of 76.94% and 76.73%, respectively. Men reported a screening rate of 72.75%, while the rate for women stood at 76.81%, indicating a higher propensity among women. Disaggregating the data by marital status, individuals with a spouse had a significantly higher screening rate (77.12%) compared to their unmarried counterparts. Economically active participants also showed a higher rate (76.73%) than those inactive. Rural residents had a screening rate of 75.40%, surpassing their urban counterparts.

Examining educational attainment, individuals with a college degree or higher registered the highest cancer screening rate at 78.39%. In terms of household income, the highest screening rate was observed in the upper quartile (82.09%), followed by the upper middle (75.42%), lower middle (72.26%), and the lower quartile (67.10%).

In relation to health insurance, those subscribed to employee-based health insurance plans had a screening rate of 78.04%, which surpassed rates among regional subscribers and Medicare beneficiaries. For those with private insurance, the screening rate was considerably higher at 77.92% compared to those without.

Table 2 presents anthropometric data, highlighting that participants who underwent cancer screening had an average waist circumference of 84.58 cm, slightly less than the 85.95 cm of those who did not. Among those screened, the group with a normal BMI was predominant at 77.83%, whereas the non-screened group had the most individuals in the underweight category, with a percentage of 37.84%.

### 3.2. Cancer Screening in Relation to Lifestyle and Health Status

Table 3 illustrates the cancer screening rates contingent upon the lifestyle and health status of the study subjects. Statistically significant disparities in cancer screening rates were observed among groups based on variables such as self-perceived health status, heavy alcohol consumption, smoking habits, frequency of walking (at least 30 min, 5 days a week), engagement in moderate physical activity, history of cancer diagnosis, presence of multiple primary cancers, existing comorbidities, and CCI scores.

When evaluating self-perceived health status, 75.57% of the cancer-screened subjects reported good health, and 76.30% reported moderate health. These percentages were elevated when compared to the non-screened group, which reported rates of 24.43% (good) and 23.70% (moderate). Heavy alcohol drinkers among the screened group accounted for 70.21%, a figure that is lower than the 75.57% of the non-drinkers group. Similarly, 67.59% of the screened subjects were current smokers, a rate less than the 76.27% of individuals who did not currently smoke. Regarding physical activity, 78.37% of the screened subjects walked regularly (at least 30 min, 5 days a week), surpassing the 72.69% of the non-walking group. Moreover, 82.15% of the screened subjects engaged in moderate physical activity, a significant rise compared to the 72.75% in the no-moderate-physical-activity group.

A history of cancer diagnosis was slightly higher in screened subjects at 76.64% compared to 74.87% in the group without a history of cancer diagnosis. The occurrence of multiple primary cancers was also higher in the screened group, with 76.09% having one cancer and 90.61% having two cancers. In contrast, the rates were 23.91% (one cancer) and 9.39% (two cancers) in the non-screened group. Pre-existing comorbidities were more prevalent among the screened subjects, with 77.79% having two chronic diseases and 75.05% having three or more, compared to 22.21% and 24.95% in the non-screened group, respectively.

Table 4 delves into the dietary habits of the study subjects. Moreover, 76.00% of the screened subjects reported having breakfast three to four times a week. In contrast, 32.60% of the non-screened subjects frequently skipped breakfast, and 25.21% had breakfast once or twice a week. Eating out patterns revealed that 75.93% of the screened subjects dined out at least daily, and 76.82% did so 1–6 times a week compared to 24.01% and 23.18% in the non-screened group. Individuals who dined out less than once a week were the most common at a rate of 29.03% in the non-screened group. Furthermore, 80.44% of the screened subjects followed dietary therapies, while in the non-screened group, 19.56% of individuals followed dietary therapies, which was less than the 27.21% of individuals who did not follow dietary therapies. Lastly, 78.76% of the screened subjects consumed dietary supplements compared to 31.97% of individuals in the non-screened group not consuming dietary supplements and compared to 21.24% of individuals consuming dietary supplements.

### 3.3. Variation in Cancer Screening Utilization Based on Income Bracket

Table 5 presents a discernible variation in cancer screening rates based on household income tiers, segmented into categories of self-funded cancer screening, partially subsidized screening, and fully subsidized screening.

Analyzing the cancer screening occurrences over the previous two years, the highest rate was observed in the fourth quartile (upper income bracket) at 82.09%. This was followed by 75.42% in the third quartile, 72.26% in the second quartile, and 67.10% in the first quartile (lowest income bracket). Focusing on screenings that were entirely self-funded, the screening rate followed a descending pattern in line with the income quartiles: 14.87% for the fourth quartile, 11.51% for the third, 9.47% for the second, and 7.17% for the first.

Interestingly, the first quartile of household income registered the lowest self-funded cancer screening rate at 21.96%. Conversely, when considering screenings facilitated by the National Health Insurance Service and those that were entirely free, the first quartile of household income showcased the highest uptake at 49.63% and 0.74%, respectively.

### 3.4. Determinants of Cancer Screening Utilization Stratified by Income Levels

To elucidate the specific factors impacting cancer screening utilization across different income levels, a logistic regression analysis was performed segmenting the participants based on their income strata (Table 6). For the first quartile (lowest income group), most determinants, barring cancer diagnosis, notably influenced the propensity to utilize cancer screening.

When analyzing the age demographic, individuals aged 50–64 were 1.87 times more likely to opt for cancer screening compared to the 40–49 age bracket. Females demonstrated a 1.38-fold higher likelihood of utilizing cancer screening than males. Having a spouse increased the likelihood by a substantial 4.02 times, while economically active individuals were 1.44 times more inclined to use cancer screening compared to their non-active counterparts.

Rural residents were 1.18 times more prone to screening compared to city dwellers. The inclination towards screening increased significantly by 1.83 times among middle school graduates compared to those with only an elementary education. Comparatively, individuals under the National Health Insurance Service umbrella had a higher propensity for screening: 2.67 times for employee subscribers and 2.06 times for local subscribers. Enrollment in private insurance amplified the likelihood by 2.84 times.

Diet and health behaviors also played a role. For instance, individuals with a normal BMI, overweight, and obese status were 3.92, 5.20, and 3.61 times, respectively, more inclined to be screened than the underweight group. A self-assessed “average” health status increased the likelihood by 1.18 times compared to a “good” health perception, though this dropped to 0.86 times for those perceiving their health as “poor”. High-risk drinkers were 0.67 times less likely, and smokers 0.60 times less likely, to use cancer screening compared to their non-consuming counterparts. Regular walkers (30+ min, five times a week) and those engaging in moderate-intensity physical activities were 1.37 and 1.44 times, respectively, more predisposed towards screening.

For participants with a diagnosis of multiple cancers, having one coinciding cancer showed a 1.10 times higher likelihood of screening compared to none, but this dipped to 0.33 times for those with two coinciding cancers. A rising count of chronic diseases correspondingly increased screening likelihood. However, with higher Charlson Comorbidity Index (CCI) scores, the likelihood of screening decreased. Dietary habits influenced outcomes too: reduced breakfast frequency led to a diminished screening likelihood. Conversely, a lower frequency in eating out elevated the likelihood by 1.40 times. Participants undergoing dietary therapy or consuming dietary supplements were markedly more inclined, with the latter group being 2.27 times more likely to engage in screening.

### 3.5. Nutrient Intake Analysis Based on Cancer Screening Status

The nutritional status of individuals undergoing cancer screening was explored by analyzing their average daily nutrient intake (Table 7). Among the entire participant cohort, those who underwent cancer screening typically consumed greater quantities of most nutrients, with the exceptions being carbohydrates (*p* < 0.0001), thiamine (*p* < 0.0001), and niacin (*p* = 0.0033).

Subsequently, to evaluate the conformity to the Korean nutritional intake standards, for subjects aged between 50 and 64, measures like NAR, MAR, and INQ were employed. Table 8 illustrates the disparities in NAR and MAR values between individuals who had undergone cancer screening and those who had not. Individuals who had participated in cancer screening displayed superior NAR and MAR metrics for almost all nutrients. Yet, the distinctions were not statistically significant for niacin, calcium, and iron. Moreover, the INQ was further assessed to identify variations between the two groups (Table 9). Notably, those not participating in cancer screenings exhibited significantly lowered INQ values for calcium (*p* = 0.0014).

As detailed in Table 10, after adjusting for age and gender, the MAR values were found to be significantly higher, by a factor of 1.145, in individuals who underwent cancer screening compared to those who did not, with a 95% confidence interval (CI) of 1.117–1.173. Similarly, the INQ values showed a significant rise, being 1.179 times higher in participants than in non-participants, with a 95% CI of 1.150–1.209. Furthermore, these findings remained consistent after additional adjustments for education and family income. In this context, the MAR values for individuals who received cancer screening were 1.092 times greater (95% CI = 1.065–1.119) and INQ values were 1.125 times higher (95% CI = 1.150–1.209) compared to those who did not participate in cancer screening.

## 4. Discussion

This study examined the potential socioecological factors influencing cancer screening rates using data from the 2019 KNHANES. Furthermore, it compared nutrient intakes and dietary quality based on the Korean Dietary Reference Intakes (KDRI) between cancer screening users and non-users in the age group of 50–64, which has the highest cancer screening rate. From a detailed evaluation of cancer screening practices across various demographic and socioeconomic strata, we identified critical associations with cancer screening uptake. Lifestyle and health habits—specifically, alcohol consumption, physical activity, and eating patterns—significantly sway cancer screening participation. Those with healthier habits tend to undergo more screenings, underscoring the link between health awareness and proactive health actions [42,43,44,45,46].

Our findings suggest that having a spouse correlates with a higher likelihood of early cancer screening, likely because family members often encourage screening and offer social support. Previous studies support this [30]. Moreover, higher educational levels lead to increased cancer screening as education often determines health-related knowledge and attitudes [47]. Similarly, income levels play a role: individuals with higher incomes are more inclined to use cancer screening services, echoing prior findings [18]. Having private insurance also boosts the chances of utilizing these services, presumably due to reduced out-of-pocket expenses. It is notable that in systems where public and private insurance coexist, private insurance substantially influences cancer screening decisions. Prior research has reported higher screening rates among those with private insurance than those without [48].

Furthermore, our results shed light on the intricate links between lifestyle, socioeconomic markers, nutritional intake, and cancer screening behavior. Individuals who utilized cancer screening typically exhibited healthier physical metrics—they were taller, weighed less, and had a more optimal waist circumference. The BMI distribution also indicated a prevalence of those with normal or slightly overweight readings. Notably, those with moderate to good health habits had higher screening rates, suggesting heightened health awareness in this group [49,50]. In contrast, those with riskier habits, like excessive drinking or smoking, showed reduced screening rates. This can be attributed to a lack of health awareness or a general disregard for preventive measures [51,52,53,54,55,56]. Previous studies offer mixed findings on the relationship between health behaviors such as smoking, drinking, exercise, and cancer screening [47,49,50,57,58].

**Figure 2:**
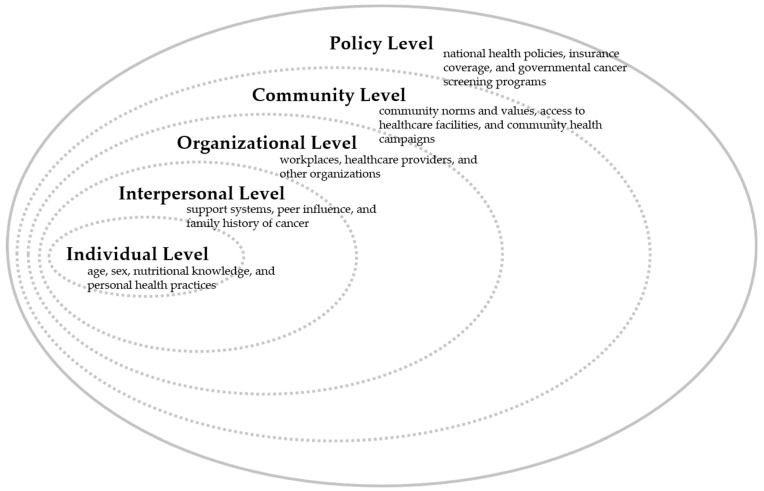
Socio-ecological framework of factors influencing cancer screening behaviors.

In the context of these findings, our study utilizes a socio-ecological model to further dissect the variables influencing cancer screening behaviors. As illustrated in Figure 2, this model categorizes factors into multiple levels: individual, interpersonal, organizational, community, and policy. Each level provides a nuanced view of the determinants influencing cancer screening behavior. Individual-level factors include personal health practices and beliefs, while interpersonal factors encompass family and social networks that may encourage or deter screening. Organizational factors relate to workplace or organizational culture and its impact on health behavior. Community factors cover the broader social and environmental conditions, and policy factors involve the health policies and systems that govern access to and the delivery of cancer screening services. This comprehensive framework allows for a more holistic understanding of the diverse influences on cancer screening uptake.

Recent studies have further underscored the significance of physical activity in modifying the course of diseases, particularly relevant in the context of COVID-19 and cancer [59,60]. Regular physical activity has been associated with milder courses of COVID-19 and reduced risk and improved survival rates in several types of cancer. These insights reinforce the notion that engaging in physical activities is not only beneficial for general health but also crucial in mitigating the severity and improving the prognosis of serious health conditions. Such findings complement our observation that individuals with healthier lifestyle choices, including regular physical activity, are more likely to participate in cancer screening programs.

In our study, dietary habits were closely analyzed vis-à-vis nutritional intake. We observed that regular breakfast consumption and a reduced frequency of eating out aligned with higher screening rates. Such patterns indicate a larger trend of health-conscious choices. The utilization of dietary supplements was common, possibly due to socio-economic factors. Additionally, individuals with higher household incomes had increased access to cancer screening, especially when they bore the expenses themselves. This echoes past research suggesting that those at higher socioeconomic rungs use preventive medical services more, especially when these entail additional costs [61]. Conversely, free screenings provided by the National Health Insurance Service were predominantly accessed by the lower income bracket, emphasizing the importance of subsidized healthcare and equity-driven policies [62,63,64,65,66,67,68].

From a nutritional perspective, the study unveiled an intriguing link between nutritious intake and higher cancer screening rates. Those who underwent screening typically had a more balanced diet, although there were exceptions. These patterns suggest either a proactive health approach among those with balanced diets or an adoption of healthier diets post screening.

This study acknowledges several limitations. Primarily, being dependent on the KNHANES data, there may be recall biases due to the nature of data collection through interviews. Additionally, as a cross-sectional study, it was limited in establishing the causal influence of independent variables on cancer screening utilization, and annual trends were not thoroughly examined. Moreover, a significant limitation is the generalization of the dependent variable related to cancer screening. The question “Have you undergone cancer screening in the past 2 years?” captures a broad range of screenings without differentiating by cancer sites or methods. This broad categorization overlooks the diversity in screening procedures, each with distinct methodologies, costs, and relevance to specific cancer types. Despite these limitations, our study provides foundational data for healthcare policies aimed at enhancing cancer screening rates. Recognizing the critical importance of detailed screening data, we suggest that future research with more comprehensive datasets could offer deeper insights into cancer screening behaviors. We also recommend that subsequent iterations of the KNHANES include more detailed information on cancer screening types and methods. Future research could also benefit from longitudinal studies and qualitative inquiries to better understand the factors influencing screening decisions.

In conclusion, this study underscores the need for targeted strategies to enhance cancer screening participation. We recommend the development of tailored educational campaigns that emphasize the importance of regular screening and healthy eating, especially in communities with low screening rates. Additionally, policy revisions to increase the accessibility and affordability of cancer screenings for lower income groups are essential. Collaborations between healthcare providers and community organizations can further promote screening awareness and nutrition education. Finally, the integration of technology, such as mobile health applications, could be a pivotal tool in improving awareness and adherence to screening schedules. Implementing these specific recommendations could significantly improve public health interventions and cancer screening rates.

## 5. Conclusions

The present research has provided profound insights into the patterns and factors influencing cancer screening behaviors among the studied population. A clear association between health and lifestyle habits, such as physical activity, drinking, and smoking, with cancer screening participation was observed. Furthermore, income disparities exhibited a noticeable influence on the utilization of cancer screening services. Specifically, higher household income quartiles were more inclined towards self-pay cancer screenings, while the lower income quartile leveraged the benefits of free cancer screenings more frequently.

Of particular interest was the relationship between nutrient intake and cancer screening status. Individuals who underwent cancer screening demonstrated a significantly higher average intake of most nutrients when juxtaposed with non-users. This observation underscores the potential correlation between health-conscious dietary habits and proactive participation in health screenings. An evaluation based on the Korean nutritional intake standards further amplified these findings, with the MAR and INQ values being markedly higher in those who opted for cancer screening.

The disparities revealed in this study highlight the importance of comprehensive public health interventions. There is a need to bridge the gaps in awareness and access across various socio-economic strata. Encouraging health-centric lifestyle habits, ensuring equitable access to screening services across different income levels, and promoting the importance of balanced nutrition can be pivotal in elevating the overall public health landscape. In the broader scheme of things, proactive cancer screening not only aids early detection but also emphasizes a broader commitment to holistic well-being. This research underscores the pressing need to integrate lifestyle, socio-economic considerations, and nutritional education into cancer screening advocacy efforts for a more profound public health impact.

## Figures and Tables

**Table 1:** Socio-demographic variables of the participants.

	Cancer Screening		
Variables	Yes (n = 206,189)	No (n = 68,683)	*p*-Value
Age (yrs.)	58.40 ± 11.37	60.33 ± 12.47	<0.0001 ^(1)^
Age (yrs.)			<0.0001 ^(2)^
40–49	56,565 (76.94%)	16,951 (23.06%)	
50–64	85,047 (76.73%)	25,794 (23.27%)	
≥65	64,577 (71.34%)	25,938 (28.66%)	
Sex (n (%))			<0.0001
Male	88,527 (72.75%)	33,163 (27.25%)	
Female	117,662 (76.81%)	35,520 (23.19%)	
Marital status (n (%))			<0.0001
With spouse	172,149 (77.12%)	51,084 (22.88%)	
Divorced	29,033 (68.28%)	13,487 (31.72%)	
Unmarried	5007 (54.91%)	4112 (45.09%)	
Employed (n (%))			<0.0001
Yes	128,883 (76.73%)	39,086 (23.27%)	
No	77,306 (72.31%)	29,597 (27.69%)	
Region (n (%))			0.0014
Urban	136,156 (74.81%)	45,837 (25.19%)	
Rural	70,033 (75.40%)	22,846 (24.60%)	
Education level (n (%))			<0.0001
≤Elementary school	40,800 (68.56%)	18,709 (31.44%)	
Middle school	24,613 (76.82%)	7426 (23.18%)	
High school	68,519 (75.17%)	22,634 (24.83%)	
≥College	72,257 (78.39%)	19,914 (21.61%)	
Family income level (n (%))			<0.0001
Low	35,092 (67.10%)	17,209 (32.90%)	
Middle low	51,652 (72.26%)	19,832 (27.74%)	
Middle high	51,810 (75.42%)	16,881 (24.58%)	
High	67,635 (82.09%)	14,761 (17.91%)	
Health insurance (n (%))			<0.0001
National health (local)	55,944 (70.54%)	23,363 (29.46%)	
National health (employer)	145,895 (78.04%)	41,065 (21.96%)	
Medicare	4350 (50.55%)	4255 (49.45%)	
Private insurance (n (%))			<0.0001
Yes	170,541 (77.92%)	48,314 (22.08%)	
No	35,648 (63.64%)	20,369 (36.36%)	

^(1)^ Different between two groups at α = 0.05 by ANCOVA test. ^(2)^ Different between two groups at α = 0.05 by chi-squared test.

**Table 2:** Anthropometric variables of the participants.

	Cancer Screening		
Variables	Yes (n = 206,189)	No (n = 68,683)	*p*-Value
Height (cm)	162.49 ± 8.88	162.13 ± 9.55	<0.0001 ^(1)^
Weight (kg)	63.34 ± 11.23	63.96 ± 12.53	<0.0001
Waist circumference (cm)	84.58 ± 9.29	85.95 ± 10.14	<0.0001
BMI (kg/m^2^)	23.90 ± 3.12	24.24 ± 3.73	<0.0001
BMI (kg/m^2^)			<0.0001 ^(2)^
Underweight	4849 (62.16%)	2952 (37.84%)	
Normal	80,450 (77.83%)	22,919 (22.17%)	
Overweight	54,639 (76.73%)	16,570 (23.27%)	
Obesity	66,251 (71.63%)	26,242 (28.37%)	

^(1)^ Different between two groups at α = 0.05 by ANCOVA test. ^(2)^ Different between two groups at α = 0.05 by chi-squared test.

**Table 3:** Lifestyle and health-related variables of the participants.

	Cancer Screening		
Variables	Yes (n = 206,189)	No (n = 68,683)	*p*-Value ^(1)^
Self-reported health status (n (%))			<0.0001
Good	60,669 (75.57%)	19,617 (24.43%)	
Moderate	110,607 (76.30%)	34,358 (23.70%)	
Poor	34,913 (70.36%)	14,708 (29.64%)	
Stress (n (%))			0.2226
Rarely	162,093 (74.96%)	54,137 (25.04%)	
Often	44,096 (75.20%)	14,546 (24.80%)	
Heavy alcohol drinking (n (%))			<0.0001
Yes	20,009 (70.21%)	8490 (29.79%)	
No	186,180 (75.57%)	60,193 (24.43%)	
Current smoking (n (%))			<0.0001
Yes	26,944 (67.59%)	12,918 (32.41%)	
No	179,245 (76.27%)	55,765 (23.73%)	
Walking (n (%))			<0.0001
<5 days/w	118,212 (72.69%)	44,406 (27.31%)	
≥5 days/w	87,977 (78.37%)	24,277 (35.35%)	
Physical activity (n (%))			<0.0001
Yes	54,369 (82.15%)	11,817 (17.85%)	
No	151,820 (72.75%)	56,866 (27.25%)	
History of cancer (n (%))			<0.0001
Yes	17,212 (76.64%)	5247 (23.36%)	
No	188,977 (74.87%)	63,436 (25.13%)	
Multiple primary cancer (n (%))			<0.0001
0	188,977 (74.87%)	63,436 (25.13%)	
1	16,324 (76.09%)	5130 (23.91%)	
2	888 (90.61%)	92 (9.39%)	
≥3	0 (0.00%)	25 (100.00%)	
Pre-existing comorbidities (n (%))			<0.0001
0	90,484 (74.45%)	31,059 (25.55%)	
1	55,393 (74.34%)	19,123 (25.66%)	
2	33,111 (77.79%)	9456 (22.21%)	
≥3	27,201 (75.05%)	9045 (24.95%)	
CCI scores (n (%))			<0.0001
0	160,504 (75.39%)	52,384 (24.61%)	
1	31,727 (74.20%)	11,033 (25.80%)	
≥2	13,958 (72.61%)	5266 (27.39%)	

^(1)^ Different between two groups at α = 0.05 by chi-squared test.

**Table 4:** Dietary habits variables of the participants.

	Cancer Screening		
Variables	Yes (n = 206,189)	No (n = 68,683)	*p*-Value ^(1)^
Eating breakfast (n (%))			<0.0001
5–7 times/w	158,359 (75.84%)	50,437 (24.16%)	
3–4 times/w	15,531 (76.00%)	4904 (24.00%)	
1–2 times/w	15,552 (74.79%)	5241 (25.21%)	
none	16,747 (67.40%)	8101 (32.60%)	
Eating out (n (%))			<0.0001
≥1 times/d	44,737 (75.93%)	14,178 (24.07%)	
1–6 times/w	107,526 (76.82%)	32,450 (23.18%)	
<1 time/w	53,926 (70.97%)	22,055 (29.03%)	
Diet therapy			<0.0001
Yes	64,137 (80.44%)	15,592 (19.56%)	
No	142,052 (72.79%)	53,091 (27.21%)	
Eating dietary supplements in a year (n (%))			<0.0001
Yes	140,931 (78.76%)	38,013 (21.24%)	
No	65,258 (68.03%)	30,670 (31.97%)	

^(1)^ Different between two groups at α = 0.05 by chi-squared test.

**Table 5:** Cancer screening type of the participants according to household income.

		Household Income				
Variables	Total (n = 274,872)	Lowest (n = 52,301)	Lower Middle (n = 71,484)	Upper Middle (n = 68,691)	Highest (n = 82,396)	*p*-Value ^(1)^
Cancer screening (CS) (n (%))						<0.0001
Yes	206,189	35,092	51,652	51,810	67,635	
	(75.01%)	(67.10%)	(72.26%)	(75.42%)	(82.09%)	
No	68,683	17,209	19,832	16,881	14,761	
	(24.99%)	(32.90%)	(27.74%)	(24.58%)	(17.91%)	
Self-pay CS (n (%))						<0.0001
Yes	30,676	3749	6770	7904	12,253	
	(11.16%)	(7.17%)	(9.47%)	(11.51%)	(14.87%)	
No	175,513	31,343	44,882	43,906	55,382	
	(63.85%)	(59.93%)	(62.79%)	(63.92%)	(67.21%)	
NA ^(2)^	68,683	17,209	19,832	16,881	14,761	
	(24.99%)	(32.90%)	(27.74%)	(24.58%)	(17.91%)	
Partial self-pay CS (n (%))						<0.0001
Yes	95,115	11,486	24,825	26,831	31,973	
	(34.60%)	(21.96%)	(34.73%)	(39.06%)	(38.80%)	
No	111,074	23,606	26,827	24,979	35,662	
	(40.41%)	(45.13%)	(37.53%)	(36.36%)	(43.28%)	
NA	68,683	17,209	19,832	16,881	14,761	
	(24.99%)	(32.90%)	(27.74%)	(24.58%)	(17.91%)	
National health insurance CS (n (%))						<0.0001
Yes	132,086	25,955	35,045	33,004	38,082	
	(48.05%)	(49.63%)	(49.02%)	(48.05%)	(46.22%)	
No	74,103	9137	16,607	18,806	29,553	
	(26.96%)	(17.47%)	(23.23%)	(27.38%)	(35.87%)	
NA	68,683	17,209	19,832	16,881	14,761	
	(24.99%)	(32.90%)	(27.74%)	(24.58%)	(17.91%)	
Free CS (n (%))						<0.0001
Yes	972	385	324	65	198	
	(0.35%)	(0.74%)	(0.45%)	(0.09%)	(0.24%)	
No	205,217	34,707	51,328	51,745	67,437	
	(74.66%)	(66.36%)	(71.80%)	(75.33%)	(81.84%)	
NA	68,683	17,209	19,832	16,881	14,761	
	(24.99%)	(32.90%)	(27.74%)	(24.58%)	(17.91%)	

^(1)^ Different between two groups at α = 0.05 by chi-squared test. ^(2)^ NA; non-applicable.

**Table 6:** Odds ratio of various factors on cancer screening in the lowest income level.

	Household Income Level	*p*-Value
Variables	Q1 (Lowest)	
	Adjusted OR ^(1)^ (95% CI)	
Age		
40–49	1	
50–64	1.865 (1.725–2.017)	<0.0001
≥65	1.637 (1.527–1.756)	<0.0001
Sex		
Male	1	
Female	1.383 (1.333–1.436)	<0.0001
Marital status		
With spouse	4.019 (3.694–4.372)	<0.0001
Divorced	1.973 (1.809–2.153)	0.5629
Unmarried	1	
Employed		
Yes	1	
No	1.444 (1.387–1.502)	<0.0001
Region		
Urban	1	
Rural	1.176 (1.133–1.221)	<0.0001
Education level		
≤Elementary school	1	
Middle school	1.826 (1.721–1.938)	<0.0001
High school	0.969 (0.921–1.020)	<0.0001
≥College	1.006 (0.931–1.088)	<0.0001
Health insurance		
National health (local)	2.059 (1.948–2.177)	<0.0001
National health (employer)	2.664 (2.528–2.808)	<0.0001
Medicare	1	
Private insurance		
Yes	2.839 (2.720–2.962)	<0.0001
No	1	
BMI (kg/m^2^)		
Underweight	1	
Normal	3.921 (3.505–4.386)	<0.0001
Overweight	5.197 (4.635–5.827)	<0.0001
Obesity	3.606 (3.223–4.034)	<0.0001
Self-reported health status		
Good	1	
Moderate	1.176 (1.117–1.238)	<0.0001
Poor	0.856 (0.809–0.906)	<0.0001
Stress		
Rarely	1	
Often	1.061 (1.014–1.111)	0.0109
Heavy alcohol drinking		
Yes	0.669 (0.620–0.723)	<0.0001
No	1	
Current smoking		
Yes	0.598 (0.565–0.632)	<0.0001
No	1	
Walking		
<5 days/w	1	
≥5 days/w	1.369 (1.317–1.423)	<0.0001
Physical activity (moderate intensity)		
Yes	1.444 (1.362–1.532)	<0.0001
No	1	
History of cancer		
Yes	1.054 (0.987–1.126)	0.1164
No	1	
Multiple primary cancer		
0	1	
1	1.104 (1.032–1.181)	<0.0001
2	0.333 (0.242–0.458)	<0.0001
Pre-existing comorbidities		
0	1	
1	1.238 (1.177–1.302)	0.0012
2	1.356 (1.282–1.434)	<0.0001
≥3	1.144 (1.083–0.209)	0.0943
CCI scores		
0	1	
1	0.931 (0.891–0.972)	0.8127
≥2	0.877 (0.820–0.937)	0.0043
Eating breakfast		
5–7 times/w	1	
3–4 times/w	0.445 (0.404–0.489)	<0.0001
1–2 times/w	0.587 (0.533–0.647)	0.0102
none	0.676 (0.619–0.738)	0.2321
Eating out		
≥1 times/d	1	
1–6 times/w	1.404 (0.298–1.520)	<0.0001
<1 time/w	1.086 (1.004–1.176)	0.0004
Diet therapy		
Yes	1.741 (1.666–1.820)	<0.0001
No	1	
Eating dietary supplements in a year		
Yes	2.267 (2.183–2.354)	<0.0001
No	1	

^(1)^ Adjusted for age and gender.

**Table 7:** Daily nutrient intakes of the participants.

	Cancer Screening		
Variables	Yes (n = 206,189)	No (n = 68,683)	*p*-Value ^(1)^
Energy (Kcal) ^(2)^	1912.01 ± 1.46	1867.22 ± 2.54	<0.0001
Carbohydrate (g)	287.57 ± 0.14	288.49 ± 0.24	<0.0001
Protein (g)	70.61 ± 0.04	68.79 ± 0.07	<0.0001
Fat (g)	43.54 ± 0.04	42.08 ± 0.07	<0.0001
Saturated fat (g)	13.14 ± 0.02	12.81 ± 0.03	<0.0001
Cholesterol (mg)	242.46 ± 0.37	223.76 ± 0.64	<0.0001
Fiber (g)	29.60 ± 0.02	28.29 ± 0.04	<0.0001
Sugar (g)	61.75 ± 0.07	57.33 ± 0.13	<0.0001
Vitamin A (µg RAE)	441.47 ± 0.91	392.57 ± 1.58	<0.0001
Vitamin B1 (mg)	1.17 ± 0.00	1.19 ± 0.00	<0.0001
Vitamin B2 (mg)	1.55 ± 0.00	1.49 ± 0.00	<0.0001
Niacin (mg)	12.24 ± 0.01	12.30 ± 0.02	0.0033
Vitamin C (mg)	74.06 ± 0.16	68.00 ± 0.28	<0.0001
Calcium (mg)	539.93 ± 0.58	505.96 ± 1.01	<0.0001
Phosphorus (mg)	1110.14 ± 0.56	1077.29 ± 0.98	<0.0001
Sodium (mg)	3473.83 ± 3.13	3440.66 ± 5.44	<0.0001
Potassium (mg)	3028.63 ± 2.12	2901.56 ± 3.68	<0.0001
Iron (mg)	10.34 ± 0.01	10.04 ± 0.02	<0.0001
Energy distribution			
% Carbohydrate	63.96 ± 0.02	64.87 ± 0.04	<0.0001
% Protein	15.45 ± 0.01	15.06 ± 0.02	<0.0001
% Fat	20.59 ± 0.02	20.07 ± 0.03	<0.0001

^(1)^ Different between two groups at α = 0.05 by ANCOVA test adjusted for age, gender, and energy (except energy). ^(2)^ Age and sex-adjusted least squares means (LSmeans).

**Table 8:** NAR ^(1)^ and MAR ^(2)^ of the participants aged 50 to 64 years.

	Cancer Screening		
Variables	Yes (n = 85,047)	No (n = 25,794)	*p*-Value ^(3)^
Carbohydrate ^(4)^	2.29 ± 0.00	2.31 ± 0.01	0.0001
Protein	1.36 ± 0.00	1.31 ± 0.00	<0.0001
Cholesterol	0.87 ± 0.00	0.75 ± 0.00	<0.0001
Fiber	1.33 ± 0.00	1.32 ± 0.00	0.0099
Vitamin A	0.71 ± 0.00	0.68 ± 0.00	<0.0001
Vitamin B1	1.08 ± 0.00	1.10 ± 0.00	<0.0001
Vitamin B2	1.23 ± 0.00	1.19 ± 0.00	<0.0001
Niacin	0.86 ± 0.00	0.85 ± 0.00	0.6006
Vitamin C	0.80 ± 0.00	0.75 ± 0.01	<0.0001
Calcium	0.73 ± 0.00	0.73 ± 0.00	0.3697
Phosphorus	1.67 ± 0.00	1.62 ± 0.00	<0.0001
Sodium	2.42 ± 0.00	2.39 ± 0.01	0.0003
Potassium	0.93 ± 0.00	0.90 ± 0.002	<0.0001
Iron	1.22 ± 0.00	1.22 ± 0.00	0.1218
MAR	1.25 ± 0.00	1.22 ± 0.00	<0.0001

^(1)^ NAR, nutrient adequacy ratio. ^(2)^ MAR, mean adequacy ratio. ^(3)^ Different between two groups at α = 0.05 by ANCOVA test adjusted for age and gender. ^(4)^ Age and sex-adjusted least squares means (LSmeans).

**Table 9:** INQ ^(1)^ of the participants aged 50 to 64 years.

	Cancer Screening		
Variables	Yes (n = 85,047)	No (n = 25,794)	*p*-Value ^(2)^
Carbohydrate ^(3)^	2.26 ± 0.00	2.28 ± 0.00	<0.0001
Protein	1.32 ± 0.00	1.27 ± 0.00	<0.0001
Cholesterol	0.83 ± 0.00	0.73 ± 0.00	<0.0001
Fiber	1.31 ± 0.00	1.31 ± 0.00	0.3398
Vitamin A	0.69 ± 0.00	0.67 ± 0.00	<0.0001
Vitamin B1	1.06 ± 0.00	1.05 ± 0.00	0.0019
Vitamin B2	1.20 ± 0.00	1.17 ± 0.00	<0.0001
Niacin	0.84 ± 0.00	0.84 ± 0.00	0.6840
Vitamin C	0.78 ± 0.00	0.74 ± 0.00	<0.0001
Calcium	0.72 ± 0.00	0.73 ± 0.00	0.0014
Phosphorus	1.63 ± 0.00	1.59 ± 0.00	<0.0001
Sodium	2.37 ± 0.00	2.32 ± 0.01	<0.0001
Potassium	0.91 ± 0.00	0.88 ± 0.00	<0.0001
Iron	1.20 ± 0.00	1.18 ± 0.00	<0.0001

^(1)^ INQ, index nutritional quality. ^(2)^ Different between two groups at α = 0.05 by ANCOVA test adjusted for age and gender. ^(3)^ Age and sex-adjusted least squares means (LSmeans).

**Table 10:** Odds ratio of cancer screening status and diet quality of the participants aged 50 to 64 years.

	Cancer Screening		*p*-Value ^(3)^	Cancer Screening		*p*-Value ^(4)^
Variables	Yes (n = 85,047)	No (n = 25,794)		Yes (n = 85,047)	No (n = 25,794)	
	Adjusted OR ^(3)^ (95% CI)			Adjusted OR ^(4)^ (95% CI)		
MAR ^(1)^						
4 (Highest)	1.145 (1.117–1.173)	1	<0.0001	1.092 (1.065–1.119)	1	<0.0001
INQ ^(2)^						
4 (Highest)	1.179 (1.150–1.209)	1	<0.0001	1.125 (1.097–1.153)	1	<0.0001

^(1)^ MAR, mean adequacy ratio. ^(2)^ INQ, index nutritional quality. ^(3)^ Adjusted for age and gender. ^(4)^ Adjusted for age, gender, education, and family income.

## Data Availability

The datasets for this study can be found in the Korea Centers for Disease Control and Prevention database through the following URL: https://knhanes.kdca.go.kr/knhanes/sub03/sub03_02_05.do, accessed on 12 August 2023. Individuals, including international researchers who sign up for membership, can utilize raw data from this website. However, the data access process and user manual are written in Korean.
